# Pharmacologic Anisocoria With Azelastine: The Importance of a Good Anamnesis

**DOI:** 10.7759/cureus.56649

**Published:** 2024-03-21

**Authors:** Margarida Ribeiro, Rita Teixeira-Martins, Jorge Meira

**Affiliations:** 1 Ophthalmology, Centro Hospitalar Universitário São João, Porto, PRT

**Keywords:** anisocoria, eyedrops, antihistamin, anticholinergic effects, pharmacologic anisocoria

## Abstract

Unilateral pharmacologic mydriasis is one of the differential diagnoses of anisocoria. This is a clinical case of a 37-year-old male patient admitted to the ophthalmology emergency department with unilateral mydriasis, an infrequent side effect of the antihistaminic drug azelastine. A comprehensive medical history including ocular medication was essential to avoid the need for additional tests and to exclude life-threatening conditions responsible for a similar presentation.

## Introduction

Anisocoria is a condition characterized by unequal pupil size. Its etiology is varied, ranging from benign to potentially life-threatening conditions. This phenomenon results from disturbances in the pupillary efferent pathway (sympathetic and/or parasympathetic arm). There may be an impairment in pupil dilation/ mydriasis (sympathetic response) or constriction/ miosis (parasympathetic response). When the sympathetic response is impaired, the abnormal pupil is the smaller one. Although in up to 20% of cases, it can be physiologic, life-threatening conditions such as Horner's syndrome should be excluded (a classic triad of ptosis, ipsilateral miosis, and anhidrosis). On the other hand, mydriasis results from damage or blockage of the parasympathetic response, resulting in a larger pupil. This may result from benign conditions such as Adie's pupil, traumatic mydriasis, or pharmacologic mydriasis, but a third nerve palsy should always be ruled out. However, this last cause is a rare scenario and it is usually accompanied by ptosis (on the side of the larger pupil) and/ or abnormal ocular motility. Pharmacologic anisocoria is much more common and can be caused by a myriad of medications (systemic and topical, including, ocular and nasal application), chemical substances, native medicines, or even following exposure to certain insecticides. It is a reversible (upon discontinuation of the medication) and benign condition [1]. The present case intends to highlight the importance of a thorough anamnesis, including an inquiry about topical medications, and to describe a case of pharmacologic anisocoria due to ocular azelastine. 

## Case presentation

A 37-year-old male patient was sent from a primary care unit to the ophthalmology emergency department with complaints of blurry vision and a perception of change in pupil size that had developed in the previous day. He had a history of keratoconus. There were no other significant findings in his medical history. He denied any regular medication. 

**Figure 1:**
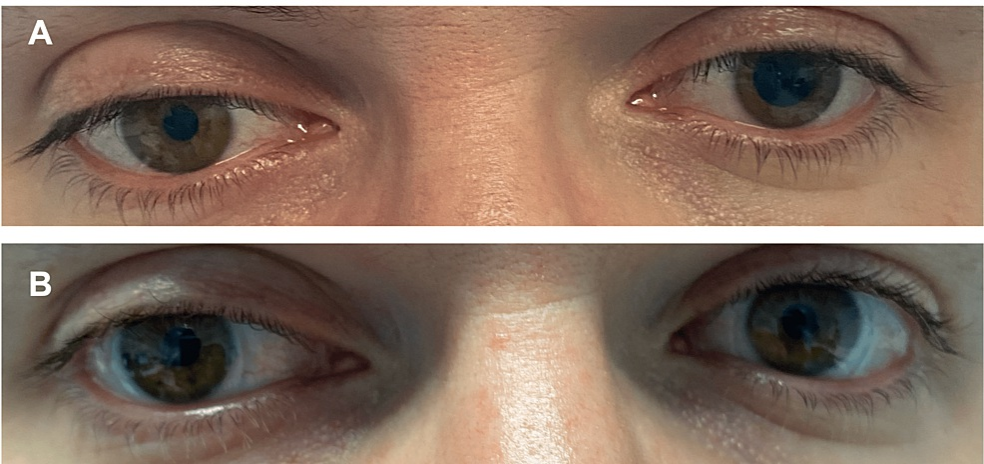
(A) Anisocoria with left pupil in full mydriasis one day after applying one drop of ocular lubricant containing azelastine. (B) Complete resolution of anisocoria 3 days after stopping this eyedrop.

Upon ophthalmological objective examination, he presented a best-corrected visual acuity of 10/10 in the right eye and 8/10 in the left eye. He presented with anisocoria (left pupil larger than the right), which seemed to be more pronounced in light. The left pupil reacted relatively poorly to both light and near stimuli. No alterations or asymmetries of the palpebral fissure were noticed, and the ocular movements were preserved. The rest of the ophthalmological examination was unremarkable (including intraocular pressure and observation of the anterior and posterior segments). Upon further questioning of his medical history, the patient admitted that on the previous day, he had complaints of itching his eye and he purchased and applied an ocular lubricant containing azelastine 0.5% in the left eye. The patient discontinued the use of this eyedrop, and when observed after 72 hours, there was a complete resolution of the anisocoria (Figure 1).

## Discussion

Pharmacologic dilation of the pupil (mydriasis) is typically characterized by poor or no pupillary constriction to light or near stimuli. Clinical differentiation from similar appearing life-threatening pathologies, most notably compressive lesions of cranial nerve III, is essential. Most commonly, pharmacologic pupillary dilation results from iris parasympathetic receptor blockade. Azelastine is an antihistaminic commonly used in allergic conjunctivitis and it is an over-the-counter drug [2]. It is a second-generation H1 antihistamine with a low affinity for cholinergic receptors. Although uncommon, pharmacological mydriasis is a side effect of this class of drugs [3]. In cases of anisocoria, it is crucial to inquire about all medications the patient is taking, including eyedrops, sometimes mistakenly not considered medication for the patient. A comprehensive medical history is essential in daily practice, potentially avoiding the need for additional tests, including pharmacological testing with pilocarpine in the approach of a mydriatic pupil [4].

## Conclusions

While a multidisciplinary approach to anisocoria should always be advocated, awareness among primary healthcare physicians should be encouraged in screening and guiding patients with anisocoria. A thorough medical history, including an inquiry about ocular medications, is a simple yet important aspect to always keep in mind.
